# Rotation of stress fibers as a single wheel in migrating fish keratocytes

**DOI:** 10.1038/s41598-018-28875-z

**Published:** 2018-07-17

**Authors:** Chika Okimura, Atsushi Taniguchi, Shigenori Nonaka, Yoshiaki Iwadate

**Affiliations:** 1 Faculty of Science, Yamaguchi University, Yamaguchi, 753-8512 Japan; 2 Laboratory for Spatiotemporal Regulations, National Institute for Basic Biology, Okazaki, 444-8585 Japan

## Abstract

Crawling migration plays an essential role in a variety of biological phenomena, including development, wound healing, and immune system function. Keratocytes are wound-healing cells in fish skin. Expansion of the leading edge of keratocytes and retraction of the rear are respectively induced by actin polymerization and contraction of stress fibers in the same way as for other cell types. Interestingly, stress fibers in keratocytes align almost perpendicular to the migration-direction. It seems that in order to efficiently retract the rear, it is better that the stress fibers align parallel to it. From the unique alignment of stress fibers in keratocytes, we speculated that the stress fibers may play a role for migration other than the retraction. Here, we reveal that the stress fibers are stereoscopically arranged so as to surround the cytoplasm in the cell body; we directly show, in sequential three-dimensional recordings, their rolling motion during migration. Removal of the stress fibers decreased migration velocity and induced the collapse of the left-right balance of crawling migration. The rotation of these stress fibers plays the role of a “wheel” in crawling migration of keratocytes.

## Introduction

Crawling migration plays an essential role in a variety of biological phenomena, including development^1,2^, wound healing^3,4^, and immune system function^5,6^. It has been revealed that cell migration is driven by the following two mechanisms. One is a combination of the expansion of the front by actin polymerization^7–10^ and retraction of the rear by actomyosin contraction^11^ shown in fibroblasts^12–14^, neutrophils^5,15–18^ and *Dictyostelium* cells^5,19,20^. The other is bleb-driven migration such as extension of the front cortex by strong actomyosin contraction at the rear, shown in the migrating protist *Amoeba proteus*^21,22^.

If skin is broken, epidermal cells from the sides of the wound migrate into the wound site to repair it^3^. In fish, motile cells, termed keratocytes, play this role in the wound-healing process^23^. Each cell is composed of a frontal crescent-shaped lamellipodium and a rear spindle-shaped cell body^24,25^. At the leading edge of the lamellipodium, actin polymerization pushes the front^26–29^, whereas, at the rear of the cell body, contraction of stress fibers composed of actomyosin retracts the rear^27,30–32^ in the same way as for other cell types^12–14^. Interestingly, stress fibers in keratocytes are positioned to connect the left and right focal adhesions^30,31,33–35^. The direction of the contractile forces exerted by the stress fibers, detected as traction forces applied to the substratum, are not parallel to the migration-direction but almost perpendicular to it^26,30,32,36–40^. It seems that in order to efficiently retract the rear of the cell, it is better that the stress fibers align parallel to the migration-direction. Why stress fibers in keratocytes align perpendicular to the migration-direction is therefore an interesting question.

Anderson *et al*. and Svitkina *et al*., having observed endocytosed bead movements, have suggested that the cell body rolls during migration^27,41^. However, it has not been clear which cytoskeleton induces the rotation. Here, we directly show, using sequential three-dimensional (3D) recordings, the rolling of a wheel formed of stress fibers. Cutting off the leading edge from a migrating keratocyte did not stop this rolling, indicating that this rotation is not a result of frontal actin polymerization. Removal of the stress fibers slowed the migration velocity and induced the collapse of the left-right balance of the crawling migration. The rotation of these stress fibers thus plays the role of a wheel in crawling migration of keratocytes.

## Results

### 3D configuration of stress fibers in a keratocyte

**Figure 1:**
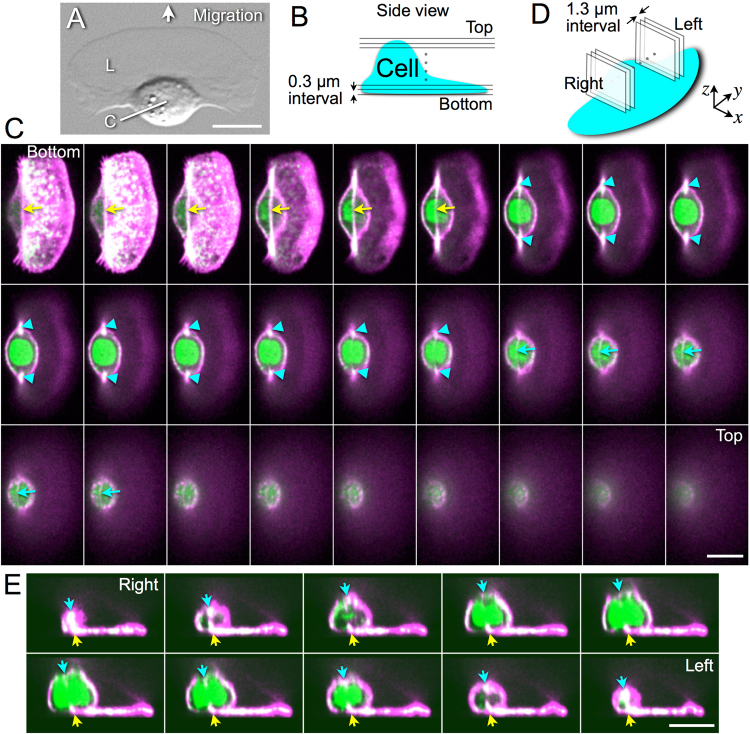
Three-dimensional configuration of stress fibers in a keratocyte. (**A**) A DIC image of a keratocyte. L: lamellipodium, C: cell body. (**B**) Positions of the optical sections shown in (**C**). (**C**) Sequential optical sections. F-actin (magenta) and myosin IIA (green). Yellow and cyan arrows: a bottom and a top stress fiber (white). Cyan arrowheads: ends of the top stress fiber. (**D**) Positions of the optical sections shown in (**E**). (**E**) Sequential perpendicular optical sections. F-actin (magenta) and myosin IIA (green). Yellow and cyan arrows: a bottom and a top stress fiber (white) correspond to those indicated by the same-color arrows in (**C**). Yellow arrows are located at the same position in each picture. Green at the center of the cell body in (**C**,**E**) represents a nucleus. Scale bars, 10 µm. Images in (**C**,**E**) are typical of 43 cells.

Keratocytes maintain their overall shape during crawling migration (Fig. 1A and Supplementary Movie S1). The lamellipodium of keratocytes is filled with a dense meshwork of thin actin filaments. In contrast, thick fibers composed of actomyosin, termed stress fibers, are present in the cell body^30,31,33–35^. To capture an accurate 3D view of the structure of the cytoskeleton, we simultaneously stained filamentous actin (F-actin) and myosin IIA in fixed keratocytes, and optical sections perpendicular to the z-axis were detected by confocal microscopy (Fig. 1B–E).

To accurately observe the configuration of the stress fibers, we arranged the optical sections from bottom to top at 0.3-µm intervals (Fig. 1B,C). Colocalization of F-actin and myosin IIA is shown as white in Fig. 1C. Stress fibers were detected in lower optical sections (yellow arrows in Fig. 1C) and also in upper (cyan arrows in Fig. 1C). Both ends of the upper stress fibers are extended into the lower optical sections (cyan arrowheads in Fig. 1C). Optical sections parallel to the x-z plane were then made at 1.3-µm intervals (Fig. 1D,E) from the reconstructed 3D images. The upper stress fiber (cyan arrows in Fig. 1E) is curved along the contour of the cell body, although the lower (yellow arrows in Fig. 1E) extends in a straight line, indicating that the stress fibers are arranged to surround the cytoplasm in the cell body. We also observed staining of the cell nucleus with myosin IIA antibody (green in Fig. 1C,E) consistent with the observations by Svitkina *et al*. and Okeyo *et al*.^27,29^.

### Rotation of stress fibers in a migrating keratocyte

In the cell body of keratocytes, F-actin and myosin IIA colocalized on the stress fibers (Fig. 1) as reported in previous studies^27,31^. Thus, next, we loaded only Alexa phalloidin into live keratocytes for high speed 3D recording. The sequential 3D images of the actin cytoskeleton in migrating keratocytes were constructed from the recorded time series of optical sections (Supplementary Movie S2). The movie clearly shows the stress fibers rotating like a wheel. The sequential 3D recordings were made not only using confocal microscopy but also by light sheet microscopy^42^.

**Figure 2:**
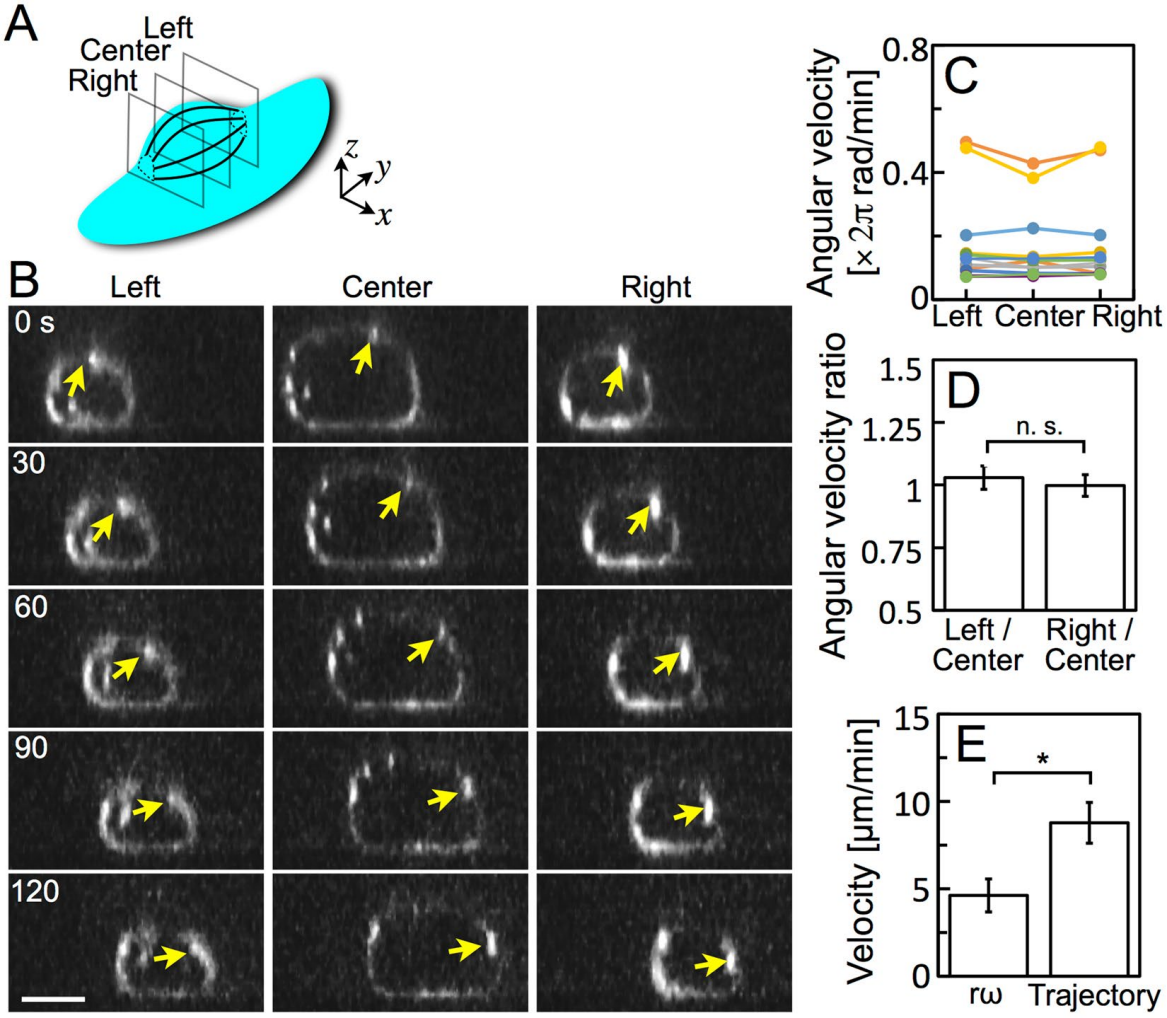
Rotation of stress fibers in a migrating keratocyte. (**A**) Positions of three optical sections, Left, Center and Right. The distance between Left and Right was defined as the length of 80% of the width of the cell body. (**B**) Rotation of stress fibers at three optical sections in (**A**), constructed from the data acquired by confocal microscopy. Yellow arrows: an identical stress fiber. Scale bar, 5 µm. (**C**) Angular velocity of the rotating stress fibers at Left, Center and Right. Twelve different cells are represented in different colors. (**D**) Angular velocity ratios of Left to Center and Right to Center calculated from the data in (**C**). (**E**) Migration velocity. rω: estimated from the product of the radius and the angular velocity at Center. Trajectory: calculated from the trajectory of the cell. Five of the twelve data in (**C**–**E**) were obtained under confocal microscopy and seven under light sheet microscopy. Error bars in (**D**,**E**) represent SEM. The *p*-values were calculated using Student’s *t*-test. *p < 0.05.

The time series of optical sections parallel to the x-z plane at the center, right and left of the cell (Fig. 2A) were made from the sequential 3D images (Fig. 2B and Supplementary Movie S3). The bright spots representing cross sections of a single stress fiber (yellow arrows in Fig. 2B) clearly rotated. The angular velocities of the stress fibers at each cross section (Left, Center and Right in Fig. 2A) were calculated from 12 migrating cells (Fig. 2C). There was no significant difference in the angular velocity among the left, center and right of individual stress fibers (Fig. 2D), indicating that the stress fibers rotate without kinking.

We estimated the migration velocity of keratocytes (rω in Fig. 2E) from the product of the radius and the angular velocity of the cell body. The estimated velocity, about 5 µm/min, was half of the real migration velocity (Trajectory in Fig. 2E) calculated from the trajectory of the center of the cell body. This result agrees with estimates made by Anderson *et al*. and Svitkina *et al*. from observations of endocytosed bead movements^27,41^, suggests that the cause of the cell body-rotation should be the rotation of the stress fibers, and indicates that the rotation of stress fibers is not the only power source of the propulsion of the cell. Combination of the stress fiber-rotation and other power sources such as frontal actin polymerization at the leading edge^9,28,43^ should realize the propulsion of the cell. We verified this assumption in the following experiments.

### Rotation of stress fibers is not a result of propulsion of keratocytes

**Figure 3:**
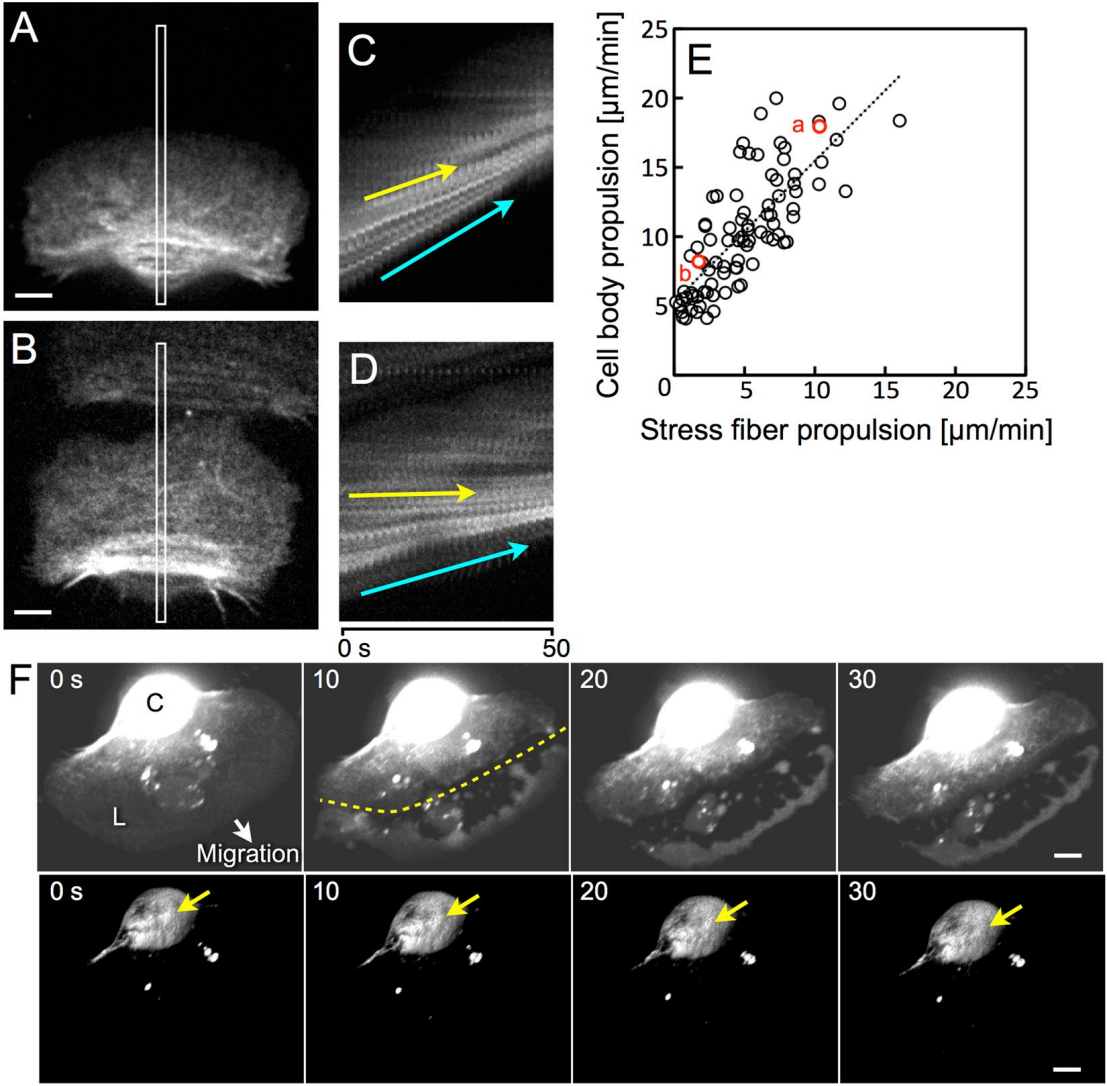
Minimal propulsion of stress fibers in a slow-migrating keratocyte and rotation of stress fibers after cutting leading edge from a migrating keratocyte. (**A**,**B**) Actin cytoskeletons at the ventral surface of a typical fast-migrating cell (**A**) and a slow one (**B**). (**C**,**D**) Kymographs constructed from image strips with width 1 µm (white rectangles in (**A**,**B**)) from sequential images taken at 2-s intervals, respectively. Yellow and cyan arrows: movements of the stress fibers and cell edge. (**E**) Relationship of the propulsion velocities between stress fiber and cell body in the laboratory frame of reference. Data of 87 cells from 14 experiments are shown. The data from (**A**) and (**B**) are indicated as red circles. Correlation coefficient (γ) was 0.78. Straight dotted line is a regression line obtained by the Least Squares method. The equation for the line is *y* = 1.02*x* + 5.29. (**F**) Sequential 3D images of stress fiber-rotation under confocal microscopy. Upper and lower images are same photos. To be able to see lamellipodium, the brightness in the upper images has been increased. From 5 to 10 s, leading edge was cut off (yellow dotted line in 10 s). L: lamellipodium, C: cell body, Yellow arrows: an identical stress fiber at upper surface of the cell body. Images in (**F**) are typical of 11 experiments. Scale bars, 5 µm.

In the case when the contribution of the stress fiber-rotation to the cell propulsion is 100% and that of other sources is 0%, the rotating stress fibers should appear to stop at the ventral surface of the cell in the laboratory frame of reference. On the other hand, when the contribution of the stress fiber-rotation is 0% and the other sources are 100%, the fibers at the ventral surface of the cell should appear to advance at the same velocity as the propulsion of the cell body. To estimate the contribution of the stress fiber-rotation to the propulsion of the cell, we compared the velocities of propulsion in detail between the stress fibers and the cell body at the ventral surface of migrating cells with various velocities (Fig. 3A–E).

The images of the actin cytoskeleton in a typical fast-migrating cell (Fig. 3A and Supplementary Movie S4) and a slow one (Fig. 3B and Supplementary Movie S5) are shown. From the sequential images at 2-s intervals, narrow regions (the white rectangles in Fig. 3A,B) were cropped and aligned sequentially to construct kymographs (Fig. 3C from A, and D from B, respectively). In fast-migrating cells (Fig. 3A), the stress fibers moved forward at the ventral surface of the cell (yellow arrow in Fig. 3C) at almost the same velocity as the cell rear (cyan arrow in Fig. 3C). However, in the slow-migrating cells (Fig. 3B), the stress fibers moved far less (yellow arrow in Fig. 3D), although the cell rear advanced (cyan arrow in Fig. 3D).

We plotted the relationship between the propulsion velocity of stress fiber and that of cell body (Fig. 3E). The slope of the regression line obtained by the Least Squares method was 1.02. When the propulsion velocity of stress fiber is zero, the velocity of the cell body is estimated from the regression line to be 5.29 µm/min. This value is almost the same as the value estimated from the rotation of the stress fibers (rω in Fig. 2E), suggesting that the rotation of stress fibers may not be merely a result of propulsion of keratocytes but contributes to it as one of the causes, at least when frontal actin polymerization is weak.

To test this possibility, we cut off leading edge from a migrating keratocyte by a glass microneedle and observed whether the rotation of stress fibers stopped or not. As expected, the stress fibers continued to rotate even after the cutting (Fig. 3F and Supplementary Movie S6).

### Role of stress fiber rotation in cell migration

**Figure 4:**
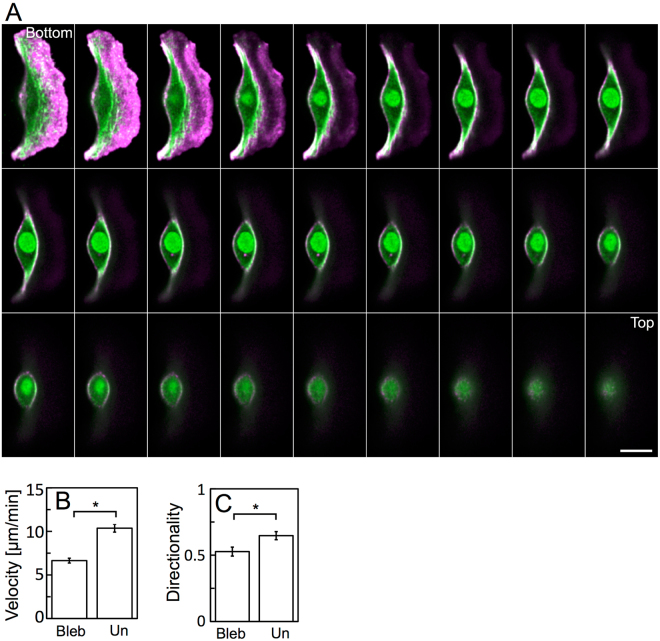
Migration of blebbistatin treated stress fiber-less keratocytes. (**A**) Sequential optical sections of a blebbistatin-treated keratocyte. F-actin (magenta) and myosin IIA (green). Areas of colocalization of F-actin and myosin IIA appear white. Green at the center of the cell body represents a nucleus. Positions of the optical sections are shown in Fig. 1B. Scale bars, 10 µm. Images in (**A**) are typical of 21 cells. (**B**) Migration velocity. Bleb: blebbistatin-treated cells (n = 43 cells from 3 experiments). Un: -untreated cells calculated from the data in Fig. 3E. (**C**) Directionality. Bleb: blebbistatin treated cells (n = 41 cells from 3 experiments). Un: -untreated cells (n = 44 cells from 3 experiments). Error bars in (**B**,**C**) represent SEM. The *p*-values were calculated using Student’s *t*-test. *p < 0.05.

If the rotation of the stress fibers contributes to the propulsion of keratocytes, removal of them should have a serious affect. Treatment with low concentrations of blebbistatin, an inhibitor of myosin II ATPase, induces disassembly of stress fibers^29,34,35^ and lateral expansion of keratocytes^31,40^. We simultaneously stained F-actin and myosin IIA in fixed blebbistatin-treated keratocytes. Their shape was slightly expanded laterally (Fig. 4A; compare with Fig. 1C). Stress fibers were disassembled in the cell body, although colocalization of F-actin and myosin IIA was detected at the rear left and right ends, and the surface of the cell body, as was the case in blebbistatin-untreated cells (Fig. 1C).

Then, to clarify whether the cell body rotates without stress fibers, we loaded only Alexa phalloidin into live blebbistatin-treated keratocytes. The sequential 3D recordings were made using confocal microscopy (Supplementary Movie S7). We detected no cell-body rotation from 17 experiments. Migration velocity of the blebbistatin-treated cells was significantly lower than that of -untreated cells (Fig. 4B).

**Figure 5:**
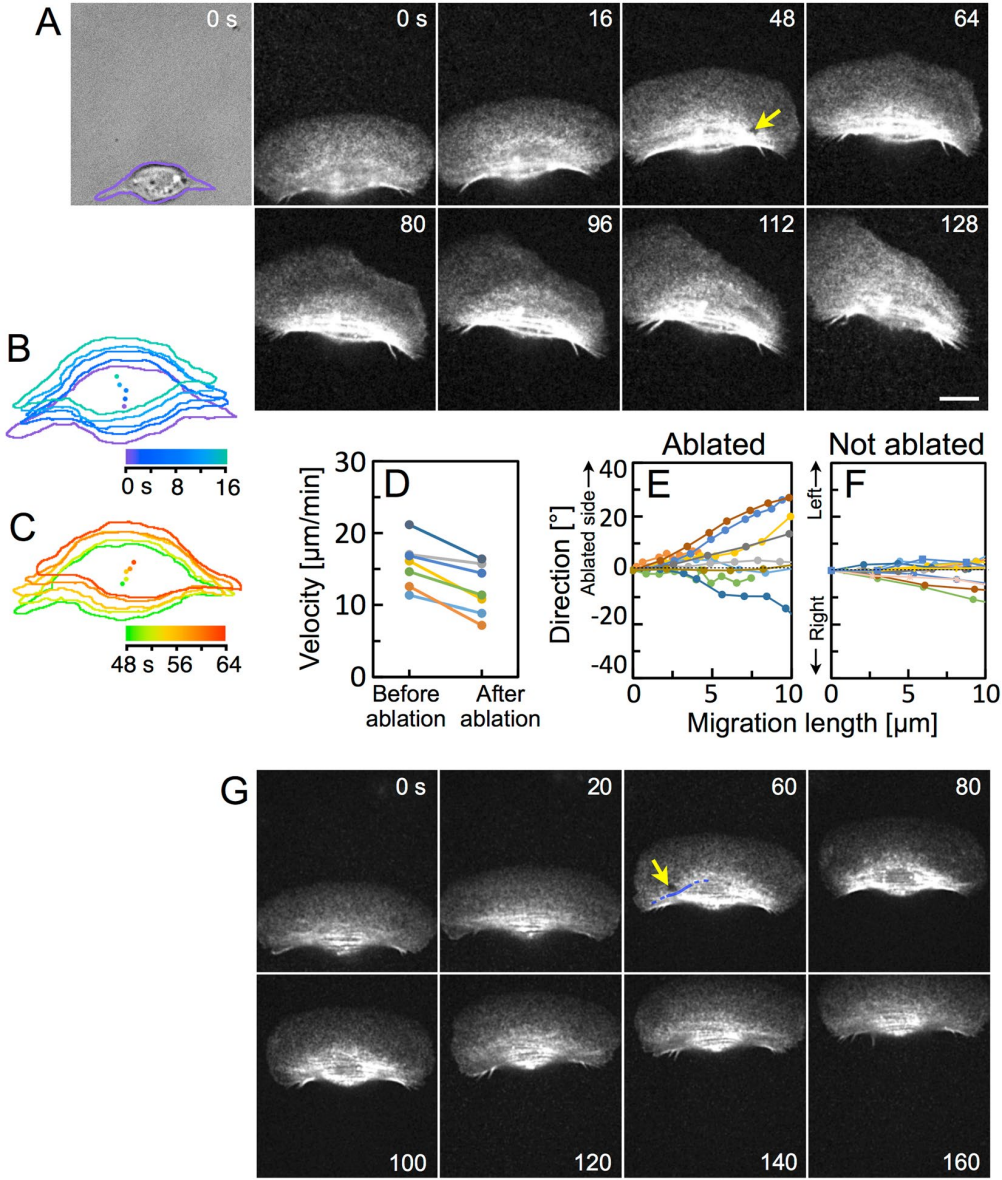
Decrease in migration velocity and destabilization of migration direction by the ablation of stress fibers. (**A**) A DIC image of a migrating keratocyte and sequential images of actin cytoskeleton in the same cell. From 17 to 32 s, parts of the stress fibers were ablated (yellow arrow in 48 s). Purple in the DIC image at 0 s: outline of the cell body. Images in (**A**) are typical of 7 experiments. (**B**,**C**) Outlines of the cell body and its centers of every 4 s before (**B**) and after (**C**) the ablation made from the sequential DIC images as 0 s in (**A**). (**D**) Migration velocities of the centers of the cell bodies before and after the ablation. (**E**,**F**) Migration directions during a 10-µm migration just after the ablation of stress fibers (**E**) and without ablation (**F**). The direction at zero migration length was defined as 0°. The value is positive when a cell turns to the same side as the ablated portion in (**E**), and when a cell turns left in (**F**). Different cells are represented in different colors (7, 10 and 13 cells in (**D–F**), respectively). (**G**) Laser beam irradiation to the outside, but to the vicinity, of the stress fibers. Sequential images of actin cytoskeleton in a migrating keratocyte. From 21 to 40 s, laser beam was irradiated to the outside of stress fibers (yellow arrow in 60 s). The stress fiber at the vicinity of the irradiation area is traced with a blue line. Images in (**G**) are typical of 5 experiments. Scale bars, 10 µm.

Blebbistatin not only disassembles the stress fibers but also inhibits cytoplasmic myosin II, such as those in lamellipodium, suggesting that some effect of blebbistatin other than disassembly of stress fibers may affect the migration. Thus, next, a portion of stress fibers at the ventral surface of a migrating cell, to which Alexa phalloidin had been loaded beforehand, was directly ablated by laser microablation (yellow arrow in Fig. 5A and Supplementary Movie S8). Outlines of the cell body traced from DIC images (Fig. 5A) and their centers every 4 s before and after ablation are shown (Fig. 5B,C). The length of the trajectory of the center appears to decrease after the ablation. As expected, the velocities of the centers obtained from 7 cells decreases after ablation (Fig. 5D). In the case when the laser beam was irradiated to the outside, but to the vicinity of, the stress fibers, it did not affect the migration of the cell (Fig. 5G and Supplementary Movie S9).

The role of wheels is not only propulsion but also steering. To reveal whether the rotation of stress fibers plays a role in steering, first, we compared the directionality of migration between blebbistatin-treated cells and -untreated cells. Directionality is expressed as the linear distance between the start and end points of migration for 30 min divided by the pathlength of the trajectory between the same points. The directionality of the blebbistatin-treated cells was significantly lower than that of the -untreated cells (Fig. 4C). We then measured the migration direction during a 10-µm migration just after the ablation of stress fibers (Fig. 5E) and compared them with those of migrating cells without ablation (Fig. 5F). The direction of migration became unstable as a result of the ablation of the stress fibers. The cells turned to the same side as the ablated portion, suggesting that the ablated parts of the stress fibers lost their function but the other side continued to advance, so the cells turned toward the ablated side.

## Discussion

A detailed observation of the actin cytoskeleton of fish epidermal keratocytes revealed that (I) stress fibers are arranged surrounding the cell body, (II) they are rotating as a single wheel even after cutting off the leading edge of the cell, (III) they do not move at the bottom when the cell migrates slowly, and (IV) removal of stress fibers decreases the migration velocity and induces the collapse of the left-right balance of the crawling migration. These results indicate that the rotation of the stress fibers plays the role of a wheel in crawling migration of keratocytes.

In the ablation experiments (Fig. 5), we can not completely exclude the possibility that the cytoskeleton other than the stress fibers destroyed by the ablation induced the collapse of the left-right balance of the crawling migration. Roy *et al*. showed that local activation of thymosin ß4, an inhibitor of actin polymerization, induced the collapse of the left-right balance of the crawling migration^44^. They concluded that thymosin ß4 destroyed the stress fiber locally, and the left-right balance collapsed. Their results also support that stress fibers are necessary for the left-right balance of the crawling migration of keratocytes.

In slow-migrating cells such as fibroblasts, it is widely accepted that there are three types of stress fibers: dorsal and ventral stress fibers that are linked to adhesion sites and transverse actin arcs that slide along dorsal stress fibers away from the front of the cell to the rear^45–48^. Whereas, fast-migrating cells such as *Dictyostelium* cells and neutrophils appear to contain no stress fibers, but rather thin F-actin bundles^16,20,49^. Fish keratocytes migrate at about 10 µm/min (Figs 2E and 4B), similar to *Dictyostelium* cells and neutrophils^50^. However, keratocytes contain stress fibers, which are typically seen in slow-migrating cells. The arrangement of the stress fibers in the cell body of keratocytes (Fig. 1) is significantly different from that of fibroblasts. Keratocyte is a unique cell that combines features of both fast and slow cell types.

The driving force of the rotation remains a mystery. We can sometimes observe stationary circular keratocytes. In contrast to motile keratocytes, arrangement of stress fibers in them are radially symmetric^51^. It is similar to the early stage of actin network self-organization in fibroblasts plated on the circular fibronectin area^52^. In permeabilized fibroblasts, myosin IIA-enriched transverse fibers move centripetally. The fibers completely diminish by the inhibition of myosin II ATPase with blebbistatin just like the stress fibers in keratocytes (Fig. 4). At the bottom of the migrating keratocyte, the stress fibers may move to the rear in the cell frame of reference with the same mechanism as the centripetal movement of transverse fibers in fibroblasts.

The left and right ends of bottom stress fibers are connected to the substratum via focal adhesions^33^. The contractile forces of stress fibers are detected as traction forces in the substratum at the trailing left and right ends^26,32,40^. At the rear end of a migrating keratocyte, detachment of both ends of a stress fiber from the substratum should take place by the contraction of actomyosin. This detachment movement of the rear stress fiber retracts the rear^27,30–32^ and may be transformed into an upward movement that becomes a driving force for the rotation of the wheel built of stress fibers. Keratocytes appear to achieve efficient migration by a combination of leading-edge extension and the rotation of the stress-fiber wheel in the cell body.

Migrating fibroblasts^53^ and keratinocytes^54^ sometimes show the shape like keratocytes spontaneously during their migration. The *amiB*^–^ *Dictyostelium* cells^55^ and the rat bladder carcinoma cells treated with imatinib^56^, an Abl family kinase inhibitor, also show the keratocyte-like shape. The arrangement of stress fibers in the tumor cells is just like that in keratocytes. The rotation of stress fibers may not be a special phenomenon characteristic to fish keratocytes but a universal migration mechanism. The observation of the rotation of stress fibers in migrating cells other than keratocytes remains of interest for future study.

Wheels are not suitable for traveling on rough surfaces. The reason that wheels did not evolve as a mobile organ in the living body may be the irregularity of natural surfaces. Fish keratocytes migrate using a wheel on the flat epithelial surfaces.

## Methods

### Cell culture

Keratocytes from the scales of Central American cichlids (*Theraps nicaraguense*) were cultured as previously described^26^. Briefly, without sacrificing the fish, a few of their scales were removed and washed in culture medium (Leibovitz’s medium: L-15, L5520: Sigma-Aldrich, St Louis, MO) supplemented with 10% fetal calf serum (Nichirei, Tokyo, Japan) and antibiotic/antimycotic solution (09366-44: Nacalai Tesque, Kyoto, Japan). The scales were sandwiched between two coverslips and allowed to adhere to one for 1 h at 23 °C. The coverslips to which the scales had adhered were kept in culture medium at 23 °C again overnight to allow the cells to spread from the scale. Cells were treated with 0.5 g/L trypsin and 0.53 mM EDTA (trypsin-EDTA, 32778-34: Nacalai Tesque) for 30–60 s to separate any cell-cell adhesions. All methods were carried out in accordance with national guidelines and the Regulation on Animal Experimentation at Yamaguchi University. All experimental protocols were approved by Yamaguchi University Animal Use Committee.

### Fixed cell staining

Fixed cell staining was performed according to the methods described previously^26,57^. Briefly, cells were fixed with 4% paraformaldehyde for 15 min, permeabilized with 0.02% Triton X-100 for 10 min, and blocked with 0.2% gelatin for 30 min. The cells were then incubated with primary antibody: rabbit polyclonal myosin IIA (1:200 dilution, M8064, Sigma-Aldrich) and Alexa Fluor 546 phalloidin (0.33 units/ml, A22283; Life Technologies, Carlsbad, CA) for 60 min. After several washes with 0.2% gelatin, the cells were incubated with secondary antibody: Alexa Fluor 488 Anti-rabbit IgG (1:2,000 dilution, A-11034, Life Technologies) for 60 min.

### Loading of Alexa phalloidin into live keratocytes

Alexa Fluor 488 phalloidin (A12379: Life Technologies) or Alexa Fluor 546 phalloidin (A22283: Life Technologies) was directly introduced into live migrating keratocytes using a self-made small-volume electroporator as described previously^58^.

### Blebbistatin treatment

(±)-Blebbistatin (13186; Cayman, Ann Arbor, MI) was dissolved at 100 mM in DMSO and then diluted 2,000 times with culture medium. This blebbistatin medium was then added to the chamber, to the bottom of which the cells had adhered, just after removal of the culture medium. After 30 min, the cells in the chamber were used for experiments without removal of the blebbistatin medium.

### Confocal microscopy

Fluorescence images of live and fixed cells were detected using an inverted microscope (Ti: Nikon, Tokyo, Japan) equipped with a laser confocal scanner unit (CSU-X1: Yokogawa, Tokyo, Japan), high speed z-axis scanner (NZ100CE, Prior, Cambridge, UK) and an EM CCD camera (DU897: Andor, Belfast, UK) through a 100 × objective lens (CFI Apo TIRF 100 × H/1.49: Nikon). For live cell recording, optical sections were recorded at 0.5-µm intervals. About 40 slices of the optical sections were recorded to construct a 3D cell image of a certain position. The exact number of the slices was dependent on the height of each cell. The time interval for recording each optical section was 56 msec. Cutting leading edge from a migrating keratocyte was performed by manipulating a glass microneedle using a micromanipulator (MO-202: Narishige, Tokyo, Japan) under confocal microscopy. The microneedle was made from a glass capillary (2–000–010, Drummond, Broomall, PA) using a pipette puller (PG-1, Narishige) and a microforge (MF-830, Narishige).

### Light sheet microscopy

The optical system of the light sheet microscope used in this study had been previously built by one of the authors, SN^42^. Light sheet illumination was applied at an angle of 45° from above the substratum on which the cells were adhered. Fluorescence images of live cells were detected using this microscope, equipped with a CMOS camera (ORCA-Flash 4.0, Hamamatsu Photonics, Hamamatsu, Japan) through a 40 × objective lens (CFI Fluor 40 × W: Nikon) which was set perpendicular to the light sheet illumination. Optical sections were recorded at 1.2-µm intervals. One hundred slices of the optical sections were recorded at 25-msec intervals to construct a 3D image of a cell a certain position.

### Laser microablation of stress fibers

Microablation of stress fibers was performed as described previously^26^ by the application of a 10-Hz pulse beam, at a wavelength of 355 nm and pulse energy of 85 µJ, for several seconds from a laser (FTSS 355-50, CryLaS, Berlin, Germany) through a 100× objective lens (CFI Plan Fluor 100×/1.30 Oil: Nikon).

### 3D image reconstruction

The consecutive images of each optical section detected by confocal microscopy or LSFM were reconstructed into 3D images using a software (FluoRender, SCI, Univ. of Utah, UT).

### Data availability

The datasets generated during the current study are available from the corresponding author on reasonable request.

## Electronic supplementary material

Movie S1

Movie S2

Movie S3

Movie S4

Movie S5

Movie S6

Movie S7

Movie S8

Movie S9

Supplementary information
