# Supplementary material for: Rotation of stress fibers as a single wheel in migrating fish keratocytes
Source: Sci Rep. 2018 Jul 17;8:10615. doi: 10.1038/s41598-018-28875-z (PMC6050267; doi:10.1038/s41598-018-28875-z)
Supplement: Supplementary file 10 — Supplementary information [file 41598_2018_28875_MOESM10_ESM.pdf]

Supplementary information

Rotation of stress fibers as a single wheel in migrating fish keratocytes

Chika Okimura[1], Atsushi Taniguchi[2], Shigenori Nonaka[2] & Yoshiaki Iwadate[1,*]

[1]Faculty of Science, Yamaguchi University, Yamaguchi, 753-8512, Japan.

[2]Laboratory for Spatiotemporal Regulations, National Institute for Basic Biology, Okazaki, 444-8585, Japan.

***Correspondence to**

Yoshiaki IWADATE, Ph.D.

iwadate@yamaguchi-u.ac.jp

Faculty of Science

Yamaguchi University

Yamaguchi 753-8512, Japan

Legends of Supplementary movies S1-S9

Supplementary movie legends

Movie S1. Crawling migration of a keratocyte. The movie depicts the same cell as that shown in Fig. 1A and is shown 30 times faster than real time.

Movie S2. Sequential 3D fluorescence images of actin cytoskeleton in a migrating keratocyte. The 3D recording was repeated at 10-sec intervals under confocal microscopy. The movie depicts the same cell as that shown in Fig. 2B and Supplementary Movie S3, and is shown 150 times faster than real time.

Movie S3. Sequential tomographic images of the actin cytoskeleton in a migrating keratocyte parallel to the x–z plane. The movie depicts the same cell as that shown in Fig. 2B and Supplementary Movie S2, and is shown 150 times faster than real time.

Movie S4. Slippage of the stress fibers at the ventral surface of a fast-migrating keratocyte. The movie depicts the same cell as that shown in Fig. 3A and is shown 30 times faster than real time.

Movie S5. Little slippage of the stress fibers at the ventral surface of a slow-migrating keratocyte. The movie depicts the same cell as that shown in Fig. 3B and is shown 30 times faster than real time.

Movie S6. Sequential 3D fluorescence images of actin cytoskeleton in a migrating keratocyte whose leading edge is cut by a glass microneedle. The 3D recording was repeated at 5-sec intervals under confocal microscopy. The movie depicts the same cell as that shown in Fig. 3F and is shown 50 times faster than real time.

Movie S7. Sequential 3D fluorescence images of actin cytoskeleton in a migrating blebbistatin-treated keratocyte. The 3D recording was repeated at 10-sec intervals under confocal microscopy. The movie is shown 150 times faster than real time.

Movie S8. Laser microablation of stress fibers in a migrating keratocyte. In our microscope, images could not be taken during ablation. The ablated portion is indicated by a yellow circle. The movie depicts the same cell as that shown in Fig. 5A and is shown 30 times faster than real time.

Movie S9. Laser beam irradiation to the outside, but to the vicinity, of stress fibers. In our microscope, images could not be taken during ablation. The irradiated portion is indicated by a yellow circle. The movie depicts the same cell as that shown in Fig. 5G and is shown 30 times faster than real time.
